# Genetic Diversity and Population Structure of the Endangered Salamander *Hynobius yiwuensis* Inferred from Mitochondrial DNA Sequences

**DOI:** 10.3390/life14060769

**Published:** 2024-06-17

**Authors:** Liangliang Zhang, Pierfrancesco Sechi, Jingbei Xie, Rui Dong, Rongquan Zheng

**Affiliations:** 1 Xingzhi College, Zhejiang Normal University, Jinhua 321100, China; liangzhang588@gmail.com (L.Z.);; 2 Independent Researcher, 07100 Sassari, Italy; 3 Zhejiang Provincial Key Laboratory of Wildlife Conservation and Utilization Technology, Zhejiang Normal University, Jinhua 321004, China

**Keywords:** *Hynobius yiwuensis*, Zhoushan Island, mitochondrial DNA, genetic diversity

## Abstract

Understanding the genetic diversity patterns of endangered species is crucial for biodiversity conservation. The endangered salamander *Hynobius yiwuensis,* endemic to the mainland and Zhoushan Island in Zhejiang, China, has suffered from sharp population declines due to habitat loss. However, the levels and patterns of genetic diversity, differentiation, and population structure of *H. yiwuensis* remain poorly understood. Here, we explored the genetic diversity and phylogeography of *H. yiwuensis* based on partial mtDNA sequences (Cytb and CO1) through 111 individuals collected from seven localities. Relatively high overall haplotype diversity (h = 0.965) and low nucleotide diversity (π = 0.013) were detected. Our results, through phylogenetic trees and haplotype network analyses, revealed two divergent haplogroups, mainland and island, and the estimated divergence time indicated they diverged ~2.44 million years ago, which coincided with the period when Zhoushan Island became separated from the mainland.

## 1. Introduction

Amphibians are globally recognized as the most endangered group of terrestrial vertebrates [1]. Climate change and other anthropic causes, such as habitat loss and fragmentation, are posing serious challenges to global amphibian biodiversity [2]. Within the family Hynobiidae, *Hynobius* is the largest genus, comprising 65 species (https://amphibiaweb.org/, accessed on 3 May 2024). The Yiwu salamander, *Hynobius yiwuensis* (Caudata, *Hynobius*), is endemic to Zhejiang, China [3]. It inhabits forests and arable fields in hilly regions and breeds in pools and small streams [4]. After breeding, the adults leave the water [5]. Recent decades have seen a sharp population decline due to habitat loss [6]. Consequently, it has been listed as a vulnerable species on the Red List of China’s Vertebrates [7], as well as a national Level Two protected species on the list of state-protected wildlife coreleased by the National Forestry and Grassland Administration and the Ministry of Agriculture and Rural Affairs (https://www.gov.cn, accessed on 1 January 2024).

So far, previous studies of *H. yiwuensis* have been limited to population investigation [8], karyotype [9,10], genetic divergence [11,12], microsatellites, and SNP markers [6,13]. Intraspecific genetic diversity tells the demographic and evolutionary histories of populations [14]. Furthermore, genetic information concerning the intraspecific genetic variability of a species is crucial to for assessing its resilience to future challenges and informing future conservation strategies. However, there remains a lack of basic genetic information for *H. yiwuensis*, such as genetic diversity, genetic structure, and genetic differentiation among different populations. Thus, it is important to elucidate the diversity and population structure of the species.

Currently, mtDNA sequences such as CO1 and Cytb are extensively employed for species identification, genetic diversity, and genetic differentiation among populations. In this study, we characterize the phylogeographic pattern and the genetic diversity of *H. yiwuensis* over most of its distribution using partial mtDNA sequences. The primary aims of our study were (a) to identify the phylogenetic relationship and genetic differentiation among geographical populations, (b) to explore which factors have shaped the patterns of genetic differentiation in *H. yiwuensis*, and (c) to provide guidance for the future conservation of *H. yiwuensis*.

## 2. Materials and Methods

### 2.1. Sample Collection and DNA Extraction

**Figure 1:**
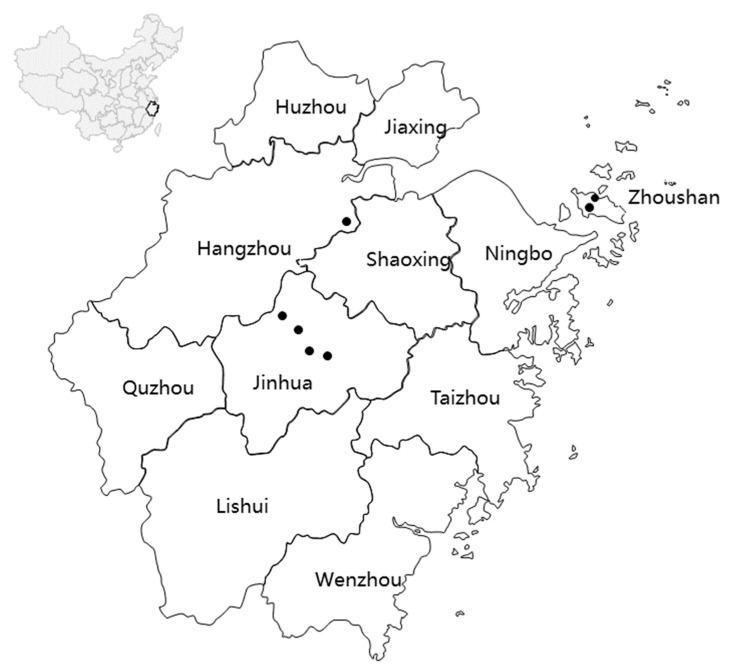
Map of Zhejiang showing sampling localities of *H. yiwuensis*.

From December 2019 to April 2021, 111 samples were collected from 7 localities covering most of the species’ distribution in Zhejiang, including 5 mainland sites (Limei (LM), Wangdao (WD), Lihuang (LH), Zhangshan (ZS), and Huantan (HT)) and 2 sites from Zhoushan Island (Ganghou (GH) and Mahuangshan (MH)) (Figure 1, Table 1). This study was approved by the Animal Research Ethics Committee of Zhejiang Normal University (ZSDW2019048, April 2019). We have complied with all of the relevant ethical regulations for animal testing and research. Samples were obtained from tail tips or toes and stored in 95% alcohol at −20 °C for DNA extraction. Individuals were carefully handled and released at the sample sites. Genomic DNA was extracted following the protocol provided with the DNA extraction kit (TIANamp Genomic DNA Kit, Sangon Biotech, Shanghai, China).

### 2.2. mtDNA Sequencing and Analysis

Two partial mitochondrial sequences, CO1 and Cytb, were amplified and sequenced for 111 samples (GenBank accession numbers PP434666—PP434791, PP440043—PP440154). The primers used for amplification were designed based on previously reported sequences of *H. yiwuensis* Cytb (JQ929941.1) and CO1 (JN165864.1) from NCBI. The PCR reactions were carried out with Taq polymerase for 35 cycles at 95 °C for 20 s and annealed at 50 °C for 20 s, followed by the extension step at 72 °C for 7 min. The products were detected by 1% agarose gel electrophoresis and sent to Sangon Biotech (Shanghai, China) for sequencing.

The sequences were aligned with Mega7 [15] and concatenated by Geneious Prime. The molecular diversity, including the number of haplotypes, haplotype diversity, and nucleotide diversity, was estimated using DnaSP 6.12.1 [16]. The maximum likelihood (ML) and neighbor-joining (NJ) phylogenetic trees were built with the MEGA7 software, using two different species (*H. amjiensis* and *H. maoershanensis)* as the outgroups. The best-fit evolutionary model selection was inferred with Phylosuite [17]. The haplotype network was built with PopArt [18]. The Bayesian skyline map (BSP) implemented in BEAST was used to estimate the historical demographic changes in the species. The divergence times were estimated using BEAST v1.8.4 [19], assuming a mutation rate of 0.68% per My, according to Yoshikawa et al. [20]. We implemented an HKY+F+G4 model selected by Phylosuite [17], using an uncorrelated lognormal relaxed molecular clock and a constant-size coalescent model as tree priors, with a total of 100 million generations. Burn-in (10%) and convergence (ESS > 200) were checked with TRACER 1.7 [21].

## 3. Results

### 3.1. Population Genetic Diversity and Differentiation of mtDNA

The number of haplotypes, as well as measures of nucleotide (π) diversity and haplotype diversity, are presented in Table 1. The aligned dataset (1219 bp) includes 37 haplotypes (Hap1-37) among 111 sequences, with high haplotype diversity (h = 0.965) and low nucleotide diversity (π = 0.013) across all populations. Each population had 2 to 13 haplotypes; 26 of the total number of 37 haplotypes were unique to one locality; 9 haplotypes were shared between two populations; and 2 haplotypes (Hap 12, 13) were shared among three populations. Notably, no haplotypes were shared between the mainland and island populations. The WD population harbored the highest genetic diversity, with the greatest number of haplotypes (n = 13) and the highest measures of haplotype (Hd = 0.966) and nucleotide (π = 0.0046) diversity, as opposed to the lowest level of haplotype diversity found in the HT population (Hd = 0.667). Overall, the mainland populations showed higher genetic diversity compared to the Zhoushan Island populations (Table 1).

### 3.2. Population Genetic Diversity and Differentiation of mtDNA

**Figure 2:**
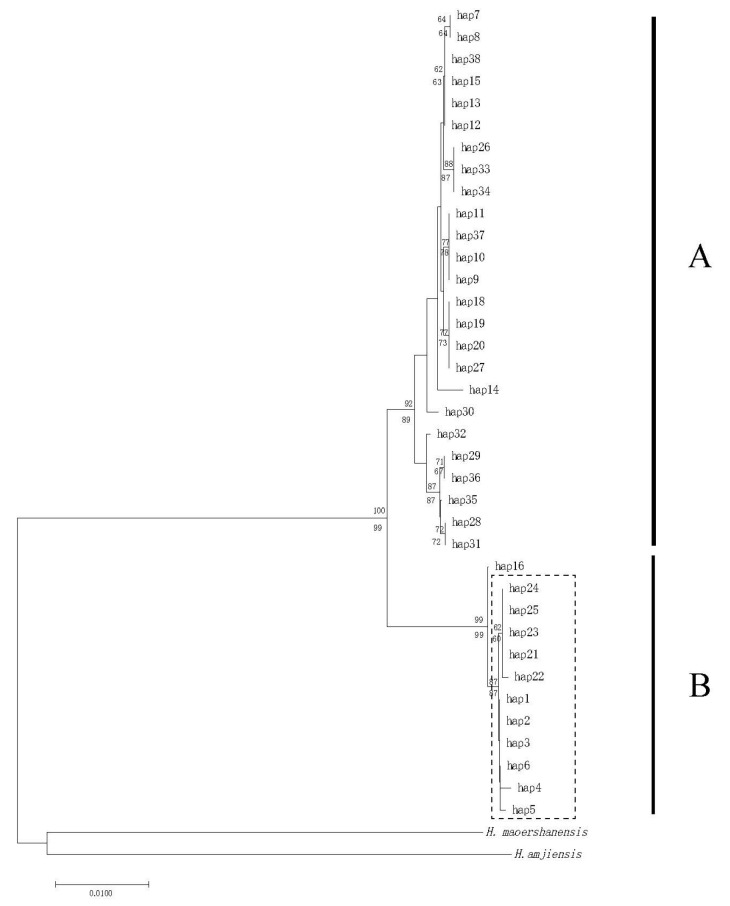
Maximum likelihood (ML) tree of *H. yiwuensis*. Numbers at the branches represent bootstrap values. A: mainland populations, B: Zhoushan Island populations.

**Figure 3:**
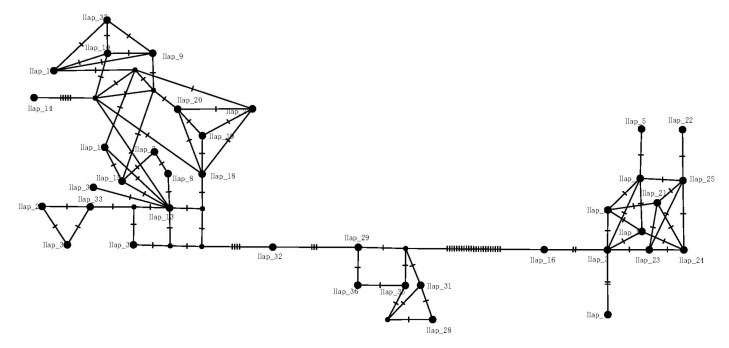
Haplotype network of *H. yiwuensis*.

The ML and NJ phylogenetic analyses based on CO1+cytb sequence fragments yielded quite similar topologies. Thus, only the ML tree is shown in Figure 2. All *H. yiwuensis* haplotypes formed a monophyletic group (ML bootstrap value: 100%; NJ bootstrap value: 99%). Within *H. yiwuensis*, two highly supported, geographically structured haplogroups (A and B) were recovered. Clade A includes all mainland populations with strong statistical support (92%, 89%). Clade B primarily consists of specimens from Zhoushan Island, except for one (hap 16, belonging to LM) from the mainland. Additionally, Zhoushan Island’s haplotypes form a monophyletic group with high support (dash-line box, 87%, 87%). A clear geographic pattern was also observed in the network, as the mainland and island populations are separated by a large number of mutation steps (Figure 3). A total of nine haplotypes (hap 1, 2, 4, 5, 21–25) are unique in the GH and MHS populations, respectively, and hap 3 and hap 6 are shared between the GH and MHS populations. That means that a total of 11 haplotypes are endemic to Zhoushan Island’s populations.

### 3.3. Demographic History and Divergence Time

**Figure 4:**
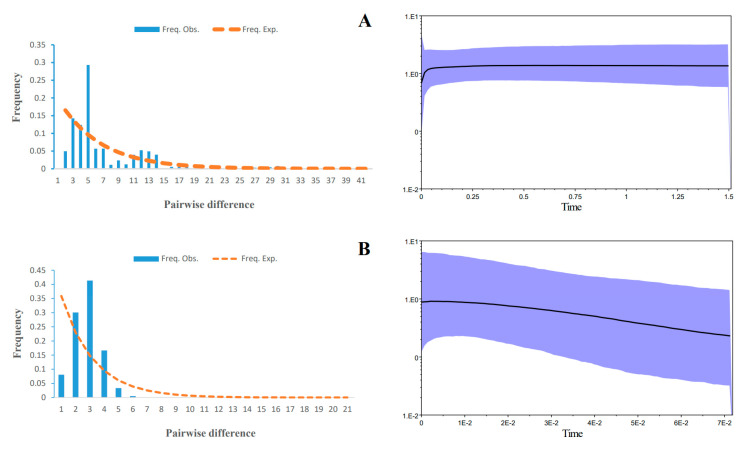
Mismatch distribution and Bayesian skyline plots for (**A**) mainland populations and (**B**) Zhoushan Island populations.

Concerning the mismatch distribution and skyline demographic reconstruction, the island and mainland populations showed different historical scenarios. The mismatch analyses for the mainland populations showed a bimodal profile (Figure 4A), which might have resulted from either a constant or declining population. In contrast, the mismatch distribution was unimodal for the island populations (Figure 4B), suggesting population expansion. Furthermore, the skyline plot suggests that the effective population sizes of Zhoushan Island’s populations gradually increased slightly (from 0.07 to 0.01 Mya ago, Figure 4B), as opposed to a long period of stable historical population size followed by a recent decreasing trend until 0.05 Mya ago, as observed in the mainland populations (Figure 4A).

**Figure 5:**
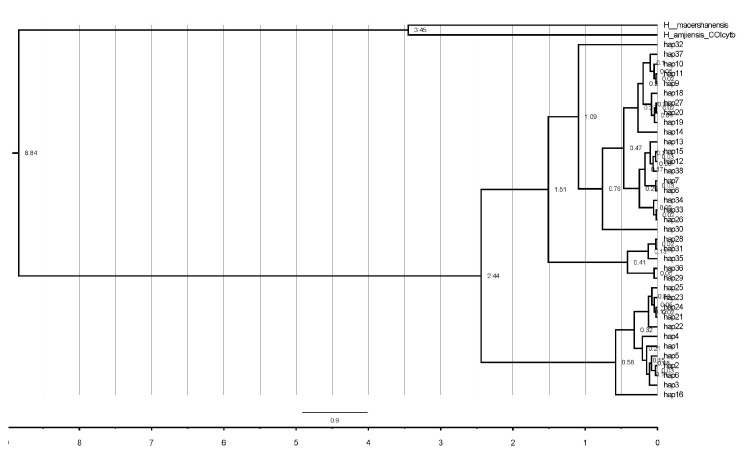
Divergence time estimation of *H. yiwuensis*. Numbers at the branches represent the node ages (Mya).

The divergence time estimates (based on a mutation rate of 0.64% per My per lineage) [17] indicate that *H. yiwuensis* originated ~2.44 million years ago, diverging into two major branches, mainland and island, with one haplotype (hap16 from the mainland) mixed within the island branch (Figure 5). Notably, the island group divided into two subgroups ~0.32 million years ago (Mya), geographically corresponding to the GH and MHS populations.

## 4. Discussion

### 4.1. Genetic Diversity

The results of our study show relatively high haplotype diversity and low nucleotide diversity across all populations. This trend is also observed in other endangered salamander species such as *Pseudoeurycea robertsi* in Mexico [22], *Neurergus derjugini* in Iran and Iraq [23], and *Pachyhynobius shangchengensis* in China [24]; thus, this is more than just a regional phenomenon. However, the genetic diversity values reported here were high compared to *H. amjiensis*, an endangered *Hynobius* species that is also found in a single locality in Zhejiang [25]. In our study, the mainland populations displayed overall higher genetic diversity than the insular populations. This pattern was also observed in *H. quelpaertensis*, a salamander on the Korean Peninsula [26]. Generally, mainland populations usually display higher genetic diversity than island populations [27].

### 4.2. Phylogenetic Inference and Genetic Structure

According to the phylogenetic and haplotype network analyses, we found two major distinct clades (A and B) primarily corresponding to the specimens from the mainland and Zhoushan Island (except for one haplotype distributed on the mainland), respectively. Our results based on partial mtDNA data are consistent with Fu et al. [12]. Both findings confirm the mainland–island phylogeographic structure of *H. yiwuensis*.

Notably, there is no haplotype shared between the mainland and island populations, with nine haplotypes being unique to the populations on Zhoushan Island. This distinctiveness was observed not only genetically but also morphologically between the island and mainland populations by Ma et al. [28], though the authors identified *H. yiwuensis* as *H. chinensis*. Collectively, these findings indicate that the island population has likely followed a distinct evolutionary path for a considerable amount of time.

### 4.3. Phylogeographic Inference

Islands act as natural barriers, profoundly shaping the genetic landscape of species. Land-bridge archipelagoes offer ideal models in order to potentially identify long-term effects on genetic variability in wild populations.

Our mismatch distribution analyses and skyline demographic reconstructions reveal distinct historical demographic patterns between the island and mainland populations. The analysis shows the island populations undergoing a gradual increase from 70,000 to 10,000 years ago, as opposed to historical stability and an ensuing decline in the mainland populations until 50,000 years ago (Figure 4A). These findings are strong support for the hypothesis of divergent demographic histories of the island and mainland populations, reflecting the impact of geographical isolation and environmental changes on population dynamics.

Zhoushan Island, an extension of the Siming Mountains, became geographically isolated from the mainland around 2 Mya due to rising sea levels [26]. This geological event corresponds to our divergence time estimates, which indicate that the principal clades (mainland and island) split from one another ~2.44 Mya. This suggests a significant impact of geographical isolation on the evolutionary trajectory of *H. yiwuensis*, although further, extended sampling from the Zhoushan Archipelago is needed to provide more evidence. Furthermore, future research efforts may explore the signatures of these diversity patterns on nuclear genes in order to give stronger support to the isolation hypothesis of the island populations.

### 4.4. Conservation Implications

Our historical demographic analyses reveal different historical scenarios between the two populations. Although the effective population sizes of the island populations underwent a continuous, albeit slight, increase, the mainland experienced a recent decline in population size. The limited distribution area (<20,000 km^2^) (AmphibiaWeb. <https://amphibiaweb.org> University of California, Berkeley, CA, USA. Accessed on 13 February 2024) and the recent sharp population decline due to habitat loss [4,6] corroborate the classification of this species as rare and endangered. Consequently, it has been recently included as a Level Two protected species on the list of state-protected wildlife by the Chinese government. However, as of today, its conservation status is still considered of Least Concern (IUCN, 2020). Based on our study results spanning its limited distribution area and its sharp decline in numbers, we suggest the species’ IUCN Red List conservation status should be changed to Vulnerable. Concerning the island population, although our skyline analysis indicates a recent slight increase in population size, we consider that given its lower genetic diversity and habitat, it should equally be targeted by consistent conservation efforts, as island populations face significantly higher extinction risks than mainland populations [27].

## Figures and Tables

**Table 1:** Sample information and genetic diversity of 7 *H. yiwuensis* sampling sites.

Locality	Site	N	nHT	Hd	π
LM	119°51′ E, 29°19′ N	14	6	0.736	0.0055
WD	119°55′ E, 29°18′ N	21	13	0.933	0.0046
LH	119°46′ E, 29°40′ N	20	8	0.884	0.0023
ZS	119°79′ E, 29°24′ N	18	9	0.908	0.0035
HT	120°18′ E, 29°57′ N	3	2	0.667	0.0011
Mainland		76	27	0.950	0.0043
GH	122°05′ E, 30°06′ N	13	8	0.910	0.0014
MH	122°06′ E, 30°08′ N	22	9	0.879	0.0012
Island		35	15	0.919	0.0015
Total		111	37	0.965	0.0130

## Data Availability

Data can be found in GenBank accession numbers PP434666—PP434791, PP440043—PP440154.
